# From photons to big-data applications: terminating terabits

**DOI:** 10.1098/rsta.2014.0445

**Published:** 2016-03-06

**Authors:** Noa Zilberman, Andrew W. Moore, Jon A. Crowcroft

**Affiliations:** Computer Laboratory, University of Cambridge, Cambridge, UK

**Keywords:** computer architecture, networking, interconnect, performance guarantees

## Abstract

Computer architectures have entered a watershed as the quantity of network data generated by user applications exceeds the data-processing capacity of any individual computer end-system. It will become impossible to scale existing computer systems while a gap grows between the quantity of networked data and the capacity for per system data processing. Despite this, the growth in demand in both task variety and task complexity continues unabated. Networked computer systems provide a fertile environment in which new applications develop. As networked computer systems become akin to infrastructure, any limitation upon the growth in capacity and capabilities becomes an important constraint of concern to all computer users. Considering a networked computer system capable of processing terabits per second, as a benchmark for scalability, we critique the state of the art in commodity computing, and propose a wholesale reconsideration in the design of computer architectures and their attendant ecosystem. Our proposal seeks to reduce costs, save power and increase performance in a multi-scale approach that has potential application from nanoscale to data-centre-scale computers.

## 1. Introduction

The rapid growth and widespread availability of high-capacity networking has meant that all computers are networked and have become integral to today's modern life. From the appliances we each have in our house and office through to the high-performance computers (HPCs) of weather forecasting to the rise of the data centre, providing our favourite search engine, storage for our family photos and pivot for much corporate, government and academic computing. The modern application presumes network connectivity and increasingly the common application presumes high-performance network connectivity to ensure the timely working of each network operation.

Such demand has been central, driving both the need for networking and the approach towards the *capacity crunch* (see other publications in this discussion meeting issue). A capacity crunch that has forced photonics development and triggered, with its lower cost of repair, (gigabit) fibre to premises to become the premier standard for Internet new entrants (e.g. Google Fiber) and incumbents (e.g. Comcast and AT&T.)

Cloud usage has driven network demand, yet the cloud itself is not well defined; a cloud may equally likely be provisioned by a company as a subscription service itself or provided as a *loss leader*. Cloud-based services may present as one or more of a provision of storage, of computing resource or of specific services (e.g. database, Web-based editor). Early cloud incarnations were driven to lower capital costs by centralizing resources and based upon arrays of commodity hosts. However, cloud provision has certainly entered the *adolescent years* and, with operational costs of electricity and maintenance eclipsing those of provision, effective cloud facilities are bespoke designs that only resemble in computer architecture the commodity boxes of their ancestry. Because few organizations can do custom design and build, the cost to install a cloud system has risen dramatically as scale-out hyper-data centres recoup investment in the bespoke design and manufacture process.

In contrast with *Cloud*—an offering of services both physical and virtual—for the purposes of the discussion in this paper, the data centre is the physical manifestation of equipment whose purpose is to provide cloud services. The data centre, from the modest physical equipment provisioned within a small department machine-room to the hyper-data centres of Google, Microsoft and Facebook, represents the equipment—computers, storage and networking—upon which cloud services operate.

Cloud-centred network applications include content distribution, e.g. NetFlix and BBC iPlayer, and the more traditional Web services of commerce, targeted advertising and social networking [1,2,3]. These sit alongside the wide range of crucial applications yet run sight-unseen supporting specialist use in engineering, science, medicine and the arts. Examples include: jet turbine modelling, car safety simulations [4] and quantitative research [5] as well as sophisticated image processing common to both medicine and the arts as movie post-production processing is now commonplace. All of this is quite aside from the network applications that support our personal communications and interactions with national security.

The effect of high-performance network applications is reflected in the increasing need for network bandwidth. Cisco's Global Cloud Index [6] for 2013–2018 forecasts that global data-centre IP traffic will nearly triple (2.8-fold) over the next 5 years, and that overall data-centre workloads will nearly double (1.9-fold). For current applications, the physical server workload is predicted to rise by 44% for cloud data centres and 13.5% for other, traditional, data centres. Also, new technologies, such as the Internet of Things, will further increase the demand on networking, compute and storage resources, growing the associated data by 3.6-fold by 2018 [6]. Alongside this an expected global consumer storage requirement of 19.3 exabyte (annually) by 2018 suggests current computing architectures do not provide an answer to forthcoming challenges.

While the move to cloud-based computing may improve performance, and reduce operational costs such as power consumption, it does not solve these challenges: the power consumption is not eliminated—rather it is centralized within the data centre, and new networking traffic (that did not exist before migrating to the cloud) is generated between the user and the cloud. Furthermore, the cloud is not always suitable: from researchers in academic institutes to people concerned with their privacy in the post-Snowden era [7], many require their servers on site. Chen & Sion [8] showed that *moving to the cloud* is not always cost-effective, as appropriate cloud services are not provided for free, and depend on the skills and particulars of both the users and the applications. Even for those wishing to migrate to the cloud, yet who have only modest network requirements for each application, e.g. download speeds of up to 2.5 Mbps or a latency of up to 160 ms, such a change would currently be impossible on the existing network infrastructure of most countries [6].

Into this lacuna we propose our alternative, Computer as Network (CAN). Before we justify our approach, in §2 we describe the needs of several modern applications. Following the outline of our use-cases, we review current computer-architecture directions in §3. We criticize these directions, highlighting their limitations in §4. In §5, we outline the underlying cause for these limitations: a developing gap in the resource of network and usable computer processing. Finally, §6 outlines our proposed CAN architecture and describes the manner in which this architecture attends past limitations. §7 notes how, as an enabling technology, this work will have an impact and outline a proposed path to the evaluation prototype.

## 2. Context

A common misconception is that the storage and processing resources of a centralized cloud data centre will suffice. Yet, just as we have communication and transportation infrastructure across continents deployed at varying scales and for varying needs (e.g. a four-lane interchange on a motorway versus a mini-roundabout in a rural area), so too do we need computing as an infrastructure.

**Figure 1.**
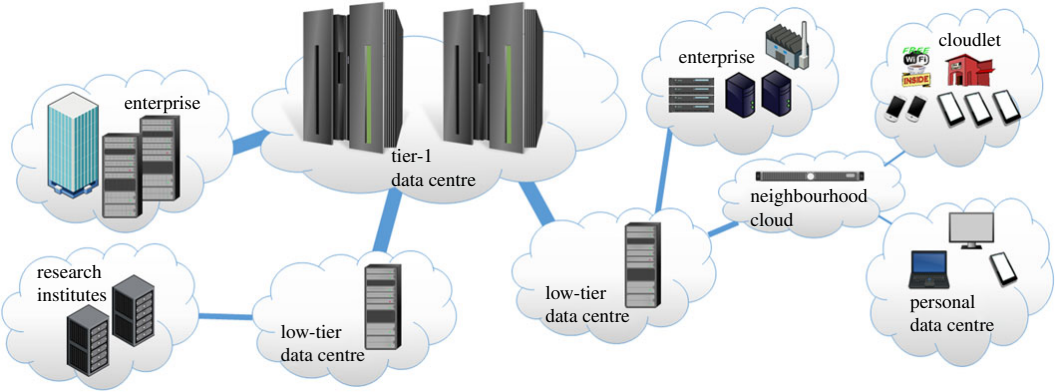
Scaling computing infrastructure: from personal computing and personal data centre to tier-1 data centres. (Online version in colour.)

Focused beyond data centres, we outline several use-cases for specific and dedicated computing infrastructure. The effect of latency, the desire for privacy and local control, and the need for availability—each motivate an infrastructure of computing provision. Just as in other types of infrastructure, there is a scaling of resources: from an unpaved road to the motorway (transportation) and from a home landline to a backbone network (communication). It is within this range of computing infrastructures that a lacuna exists: we currently step from a data centre handling petabytes per second of information to commodity servers that only handle gigabytes of data. Figure 1 depicts several use-cases that benefit from computing as an infrastructure.

Despite apparent sufficiency, the current arrangement of computers, networks and data-centre services cannot easily effect end-host latency, meet the desire for privacy and local control, or meet the need for availability. Meeting these needs, the personal data centre, suggested by Databox [9], specifically seeks to provide high availability, and local data-management and privacy controls. With sufficient computing resource, and continued advances in homomorphic encryption [10], a Databox concept could provide near perfect encryption—allowing computation by third-party services without any privacy concern.

Such sharing becomes critical as, in the manner of power and communications infrastructure, the personal data centre can serve more than a single household. Just as consumers are already familiar with being paid for returning excess solar-panel production to the grid, personal data-centre services could be provided in a similar way. The owner of a personal data centre could, just as some British Telecom users share their home wireless and extend their wireless coverage through other participating consumers' equipment, share resources from their personal data centre among other similarly minded contributors. A personal data centre that allows sufficient resource control, moderation and security is an important enabling technology.

Mobile computing has provided the spearhead use-case for cloud-based computation. It lowers handset power consumption and maximizes battery life by offloading computations. However, conducting most of the computations off the mobile device is combined with a need for a crisp user experience that requires low latency. *Cloudlets* [11] have been proposed to address the latency needs for mobile-device offload. A Cloudlet is a trusted, resource-rich, computer that is both well connected to the Internet and available for use by nearby mobile devices. Cloudlets are an interim computing level between mobile devices and full cloud services, but they also require powerful local computing. This local computing resource needs to support isolation between a large number of users. Cloudlets also require considerable processing resources in order to both offload mobile computations and provide a satisfying user experience.

Regardless of their size, enterprises are very sensitive to their computing infrastructure. Almost any company today requires significant computing resources and many are encouraged to move their computing and storage to the cloud—to data-centre services owned by other companies. Such moves may not be appropriate: a legal company has certain confidentiality constraints that force data to be on company premises, or a video post-production house must simultaneously manipulate dozens of 50 Gbps (8 k video) data streams. In each of these companies, commodity servers are insufficient for their growing needs, yet moving workloads to the cloud adds too much latency, is too expensive or is illegal. An enterprise must seek a solution that provides the high performance, reliability and resilience required for its needs, but also the ability to maintain physical access and to support the computer: that is, support a large number of users with different sets of policies, and be able to monitor their activities, debug problems and recover from failures.

Research projects where data must be physically co-located with measurement apparatus, or where it is not practical to move data to a remote facility, share much in common with the enterprise case. Furthermore, despite growing research budget pressures, research computing needs continue to grow. We offer a roadmap to high performance and strong isolation that is perfect for sharing common facilities.

The need for smaller data-centre facilities nearby to users has not escaped the notice of the larger data-centre cloud operators; and they also have a need for tiers of smaller data centres deployed closer to users. Such deployments of many thousands of small data centres at the edge of their networks further supports our need-based uses.

## 3. Trends in computer architecture

In the last decade, a revolution in computing concepts has occurred. Alas, most efforts have focused upon the economy of scale, exploiting cost advantages by expanding the scale of production. This puts data-centre computing at the focus of interest. To name just a few of these efforts, Firebox [12] is an architecture containing up to 10 000 compute nodes, each containing about 100 cores, and up to an exabyte of non-volatile memory connected via a low-latency, high-bandwidth optical switch. Firebox is focused on designing custom computers using custom chips. HP's The Machine [13] focuses on flattening the memory hierarchy using memristor-based non-volatile memory for long-term storage. The Machine also uses a photonic interconnect to provide both good latency and high bandwidth among sub-systems. This wholesale use of new technologies that provide a near-flat latency interconnect and widely available persistent memory has a large impact upon any software. Both Firebox and The Machine look at data-centre-scale computing that has requirements different from a collection of co-located servers, and thus their designs require a holistic approach that treats the entire system as a single machine [14]. However, the complexity of these new machines and immaturity of the new technologies led to limited applicability and a longer time for adoption.

Another approach that exploits the economies of scale by using commodity components is represented by RackScale [15] and the Open Compute project [16]. The RackScale architecture is usually referred to by three key concepts [17]: the disaggregation of the compute, memory and storage resources; the use of silicon photonics as a low-latency, high-speed fabric; and, finally, a software that combines disaggregated hardware capacity over the fabric to create ‘pooled systems’. The Open Compute project [16] is an open source project that seeks to design the most efficient server, storage and data-centre hardware for data-centre computing. Here every element in the data centre is based on customized boxes built around commodity components and open source software, and cannot be considered to revolutionize computing concepts. Rack scale is not well defined, it can refer to a large unit, filling part of a rack; it may also refer to a single rack [18]; and it sometimes also refers to a small number of racks [19].

Several commercial products have tried to address the growing computing needs. Examples include HP's Moonshot [20] and AMD's Seamicro [21]. These machines range in size from one to 10 rack units (being a quarter of a full rack), and sometimes contain more than 1000 cores, divided between many small server units. Unfortunately, none of these products provides a long-lasting scalable solution, as shown by HP replacing the Moonshot architecture with The Machine, and SeaMicro being cancelled by AMD [21].

Specialist HPC applications have driven a set of requirements disjoint from personal or cloud computing. These expensive single-application systems target very different types of applications and thus are outside the scope of this work.

## 4. Limitations of current-day architectures

The computing industry historically relied on increased microprocessor performance as transistor density doubled [22], while power density limits [23] led to multi-processing [24]. Common servers today consist of multiple processors, each consisting of multiple cores, and increasingly a single machine runs a hypervisor to support multiple virtual machines (VMs). A hypervisor provides to each VM an emulation of the resources of a physical computer. Upon each VM, a more typical operating system and application software may operate. The hypervisor allocates each VM memory and processor time. While a hypervisor gives access to other resources, e.g. network and storage, limited guarantees (or constraints) are made on their usage or availability.

While VMs are popular, permitting consolidation and increasing the mean utilization of machines, the hypervisor has limited ability to isolate competing resource use or mitigate the impact of usage between VMs. Beyond predictability, this also limits robustness, as machines are sensitive to the mix of loads running on top of them. Resource isolation has been the focus of considerable research over the years, with examples from the multi-processor set-up to resource isolation between VMs (e.g. [25]). Work on precise hardware communication-resource allocation has been limited in scalability or has been carried out as thought-experiments (e.g. [26]).

Resource isolation is not the only challenge for scaling computing architectures. General purpose central processing units (CPUs) are not designed to handle the high packet rates of new networks. Doing useful work on a 100 Gbps data stream exceeds the limits of today's processors. This is despite the modern CPU intra-core/cache ring-bus achieving a peak interface rate of 3 Tbps [27], and a peak aggregate throughput that grows proportionally with the number of cores. Unlike networking devices, CPUs may spend many instruction cycles per packet. Even the best current networking driver requires over 100 cycles to send or receive a packet; doing useful work is even more cycles: an application accessing an object on a small (8 Mbyte) list structure requires at least 250 cycles [28]. A data stream of 100 Gbps, with 64 byte packets, is a packet rate of 148.8 M packets per second; thus a 3 GHz CPU has only 20 cycles per packet: significantly less than required even just to send or receive. The inefficiency of packet processing by the CPU remains a great challenge, with a current tendency to offload to an accelerator on the network interface itself [29].

In-memory processing and the use of remote direct memory access as the underlying communications system is a growing trend in large-scale computing. Architectures such as scale-out non-uniform memory access (NUMA) [30] for rack-scale computers are very sensitive to latency and thus have latency-reducing designs [31]. However, they have limited scalability due to intrinsic physical limitations of the propagation delay among different elements of the system. A fibre used for inter-server connection has a propagation delay of 5 ns/m; thus, within a standard height rack, the propagation delay between the top and bottom rack units is approximately 9 ns, and the round-trip time to fetch remote data is 18 ns. While for current generation architectures this order of latency is reasonable [31], it indicates scale-out NUMA machines at data-centre scale (with each round-trip taking at least 1 μs) are not plausible, as the round-trip latency alone is many magnitudes the time-scale for memory retrieval off local random access memory or the latency contribution of any other element in the system. With latencies aggressively reduced across all other elements of in-memory architectures, such propagation delays set a limit on the physical size and thus the scalability of such an architecture.

Photonics has advanced hand in hand with network-capacity growth. However, photonics has its own limitations [32]: the minimum size for photonic devices is determined by the wavelength of light, e.g. optical waveguides must be larger than one-half of the wavelength of the light in use. Yet, this is over an order of magnitude larger than the size of complementary metal-oxide semiconductor transistors. Consequently, the miniaturization of photonic components faces fundamental limits and photonic device sizes cannot be continuously scaled in physical dimensions. Furthermore, unlike electronic devices in which the signals are regenerated at each sequential element, optical loss, crosstalk and noise build up along a cascade of optical devices, limiting scalability of any purely optical path.

Limitations are faced at several levels in the system hierarchy: from the practical limitations of physics to the increasing *impedance mismatch* between processor clock speed and network data rates. We have not even discussed the limitations of economics and politics that mean both large and low-latency (closed) data-centre facilities are rarely plausible: there is no practical real estate for a large data centre within the M25 orbital of London, UK, even if it were politically desirable. As a result, we motivate our own work to address a range of limitations from the impact of bandwidth, the desire to reduce application latency and also to be a practical and enabling effort: one that allows a decentralization of resources with the attendant advantages that bring forth power, privacy and latency.

## 5. The gap between networking and computing

The silicon vendors for both computing and networking devices operate in the same technological ecosystem. CPU manufacturers often had access to the newest fabrication processes and the leading edge of shrinking gate size, while most networking device vendors are fabless, lagging a few years behind in capitalizing on this. Yet, networking devices have shown over the past 20 years superior datapath bandwidth improvement to CPUs [32]. Furthermore, in the past 20 years, the interconnect rate of networking devices doubled every 18 months, whereas computing system I/O throughput doubled approximately every 24 months [33]. At the interface between network and processor PCI-Express, the dominant processor-I/O interconnect, the third generation of which was released in 2010, achieves 128 Gbps over 16 serial links [34]. The fourth generation—expected in 2016—aims to double this bandwidth. By contrast, Intel's inter-socket quickpath interconnect (QPI) currently achieves 96 Gbps (unidirectional). Meanwhile, networking interconnects have achieved 100 Gbps [35] and 400 Gbps [36] for several years. Furthermore, these interfaces achieve their high throughput over a smaller number of serial links: four serial links for 100 Gbps and eight serial links for 400 Gbps [37]. The limitations of existing computing interconnects vexes major CPU vendors [38].^1^

**Figure 2.**
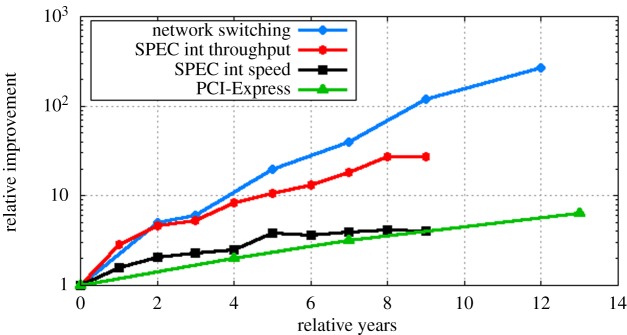
The relative improvement of computing and networking devices over the last 15 years. (Online version in colour.)

General purpose processors are extremely complex devices whose traits cannot be limited to specifications such as datapath bandwidth or I/O interconnect. Subsequently, we evaluate the performance of CPUs using the Standard Performance Evaluation Corporation (SPEC) CPU2006 benchmark [40] and contrast this with the improvement in network-switching devices and computing interconnect. To evaluate the improvement of CPUs, we consider all the records submitted to the SPEC CPU2006 database, for both integer and floating point, speed and throughput benchmarks. For every year, from 2006 to mid-2015, we select the CPU with the highest result—and since SPEC evaluates machines and not CPUs, we do that by dividing the result of each machine by the number of CPU sockets. Figure 2 shows the relative improvement of CPU speed and throughput performance over the years, growing fourfold (integer) and sevenfold (floating point) in speed and about 27-fold in throughput (both floating point and integer). For clarity, we include only integer benchmark performance improvement in the graph.

For networking devices, we observe 266-fold improvement in the throughput of standard top-of-rack Ethernet switches from 2002 to 2014 [32]. Finally, between networking and processor, the performance of the PCI-Express interconnect has improved only 6.4-fold from its introduction to the upcoming PCI-Express fourth generation. Aside from the lack-lustre improvements in interconnect standards, the other observation from figure 2 is the rate of change for each measurement. While networking device performance doubles approximately every 18 months and CPU throughput performance doubles every 24 months, the interconnect between CPUs improves at the CPU rate. Over time, we predict this lag in scaling has led to a growing gap in performance between networking and computing devices.

Networking devices have supported the need to actively differentiate traffic. Sophisticated methods for queue control exist so that network operators can deliver over the same physical infrastructure differing network traffic (e.g. video distribution, voice calls and consumer Internet traffic), each with different needs and constraints (e.g. minimal loss, constrained latency or jitter). Effort to support this means that current network silicon support controls millions of individual flows—magnitudes of more than the hundreds or thousands of VMs per host.

One of the topics that has been core to networking devices over the years is providing a predefined quality of service to users. This is what most people know as a service-level agreement (SLA). Today, Internet connectivity, television and phone services are provided to end-users over a shared networking infrastructure, and every user can opt for a different SLA for a different cost. Consequently, networking devices implement in hardware complex schemes that provide the agreed quality of service to a large number of users, such as priorities, committed and excess bandwidth, and support properties such as low latency and bounded jitter. Furthermore, these are supported for hundreds of thousands of different flows, in contrast to the hundreds or thousands of VMs per host commonly supported by VM appliances. Such network service isolation is of sufficient granularity to support the requirements identified in §4.

Not only is energy consumption and dissipation the primary force in CPU design but heat dissipation is important to ensure device performance: the features of devices are dictated by the thermal footprint. In contrast with CPUs, networking devices have managed to avoid complex dissipation systems, using only passive heat-sinks and enclosure fans. Thus, the reliability of networking systems is higher while the electrical operating costs are lower than a typical server.

To bridge the performance gap between networking and computing by capitalizing upon the promise of current networking approaches, we can draw upon the practices of network switch silicon [41] while applying the long-standing principles of stochastic networking [42]. One might envisage a stochastic treatment of transactions permitting an application of certain bounded throughput and latency for each transaction context.

## 6. Computer as Network

To address the limitations presented in §5, as a result of the limitations discussed in §4, we present a new computer architecture, dubbed CAN (Computer as Network). This new architecture borrows ideas and practices from the networking world to address the challenges presented by computing.

### (a) The CAN architecture

**Figure 3.**
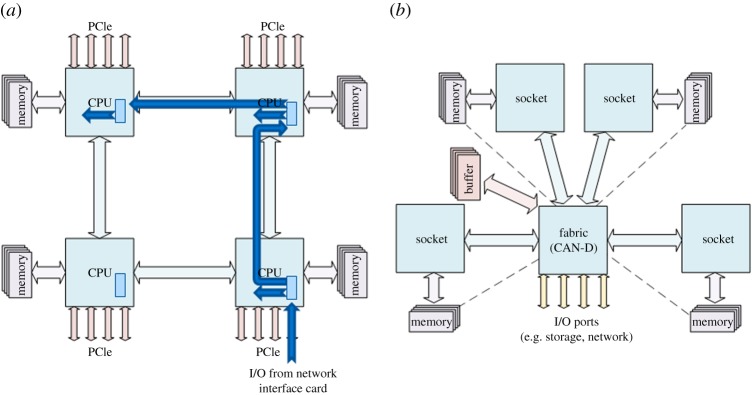
Comparative architectures: (*a*) common commodity four-socket system, (*b*) proposed CAN concept. In the common commodity system, an I/O channel is associated with a specific dedicated socket. In the CAN concept, all I/O (storage, networking, etc.) is associated with the CAN-D. (Online version in colour.)

CAN server architecture is focused on multi-core, multi-socket servers and explicitly has a networking fabric at the core of the computing device, as illustrated in figure 3. Key to this proposed architecture is that every I/O transaction among elements in the system is treated. By *transaction*, we refer to any movement of data over any interconnect within the server; in the CAN we do not differentiate transactions for networking from transactions for I/O devices from transactions for memory or inter-socket communications. In all cases, like the handling of data packets within networks, we can apply performance guarantees such as priorities, throughput guarantees and latency guarantees to each transaction. A side-effect is to minimize cross-talk between any transaction: that is, every I/O operation. This also enables us to use more efficient, network-centric, methods for moving data among the components of a computer architecture.

Figure 3 illustrates the concept of CAN, contrasting a commodity four-socket architecture with CAN. It is clear in the commodity four-socket architecture that, because I/O systems, network devices, graphics devices, etc., are each associated with a particular socket, any data generated off-socket must hop-by-hop progress through the X–Y grid of sockets, in addition to introducing variable latency; older results show [43] 150 ns variance across an eight-socket system. Such data movement will consume intermediate communications and cache resources as transactions are relayed through the CPU-based network. By contrast, the CAN approach is to use a low-overhead switching element with direct single-hop access to every socket. Even today, standard switching silicon can achieve switching delays of less than 100 ns independently of the port-count or fabric size. Now consider a CAN server in a standard (19-inch) rack width. The server contains several CPU sockets: the number of sockets can comfortably be between two and 12 sockets. This value is derived by realistic server power and space considerations. Each CPU socket is directly connected to the network fabric, which consists in turn of one or more network devices. Alongside the networking and I/O ports, the fabric device, CAN-D (CAN Device), also connects directly to all the peripherals of the server: storage devices, memory devices, graphic processing units (GPUs), etc. Finally, the CAN-D has direct access to a large quantity of its own buffering memory.

Any new transaction arriving to CAN-D, either from within the computer or externally, is mapped to a destination device (which can be any device on board), and to a flow. A flow can be destined to a process of a VM on a core within a particular CPU socket, or any other peripheral device. Each flow is assigned a set of properties, such as committed throughput, absolute priorities and bounding on latency. Each transaction can be guaranteed these properties as it is forwarded to its destination. Low-priority transactions can be stored in the CAN-D buffering, to allow transactions with higher priority to jump the queue and receive priority handling. A common network facility, CAN-D allows a transaction to be trivially sent to multiple destinations (multi-cast); such abilities would provide for easy replication and locking primitives. Finally, the CAN architecture supports multi-controller access to memory devices such as the hybrid memory cube (HMC [44]). Thus, a high-throughput flow can be written directly to the processor's attached memory without crossing the processor's interconnect nor wasting other on-socket resources. All the while, a control channel can be provided in parallel between the CAN-D and the processor, permitting permission controls and pre-emption as required.

CAN is a scale-up architecture, and as such it is important to note that CAN does not cause traffic explosion within the network fabric, as each device within the server generates transactions at a rate equal to its maximal performance, and overall the intra-server communication is designed to be of the same order as the inter-server communication. Intra-server communication scales with networking performance, and CAN-D uses common networking architectures and practices such as local buffering, thus the overall bandwidth for the CAN-D scales with commodity networking devices.

While CAN is focused on the server level, this approach can also be applied at the smaller scale processor level as well as the larger scale data-centre level. On the processor level, CAN-D can be implemented as the fabric between cores (e.g. using a photonic-based fabric layer as the bottom of a three-dimensional die-stack device). On the data-centre level, CAN can be extended to have a top of rack CAN-D or spine CAN-D interconnecting CAN fabrics across multiple servers, racks or pods. In this manner, CAN can scale-out, and not only scale-up.

In our approach, CAN opts for a non-coherent operating model, using operating systems such as Barrelfish [43] or Unikernels [45]. Support for coherency is feasible on CAN, for example to support large-coherency objects, e.g. database applications. This could be achieved by using the priority mechanics of the network fabric. However, for a highly scalable architecture that supports heterogeneous devices, where coherency cannot be supported by hardware, we claim that a software-based approach such as suggested by COSH [46] is better.

The concept of a network fabric as the core of a computer was in part inspired by the Desk Area Network (DAN) [47], a multi-media workstation that used a single, common, network interconnect among all sub-systems (CPU, memory, network, audio and video I/O devices). It also considers ideas proposed for HPCs, and adapts them to affordable commodity hardware and to computing as infrastructure use-cases.

### (b) The benefits of CAN

CAN as a scalable architecture scales with network-switching performance for inter-server transactions, and with computing-interconnect bandwidth for intra-server transactions, using existing commodity devices. The use of CAN-D bridges the performance gap between networking and computing, and provides the first entry on a roadmap for lasting performance improvement. Beyond high throughput, CAN provides the above-mentioned performance guarantees: committed and excess throughput, priorities and bounded latency. Furthermore, CAN-D is designed to support the implementation of new and emerging performance guarantees, in a manner that allows easy adoption.

CAN provides isolation between compute and I/O units, isolating processes and/or VMs as required. The isolation of resources means the completion of a job is predictable, while the computer is robust to interference: the execution time of each job no longer depends on other work running in parallel.

However, the CAN approach also has challenges: by supporting so many individually annotated transactions, the management and maintenance must not be neglected. Scalable solutions from the software-defined network community can be adopted to configure and manage flows of information in our system. We believe that we can also capitalize on the burgeoning network verification and correctness efforts to aid in CAN debugging tools from the earliest design stages.

**Figure 4.**
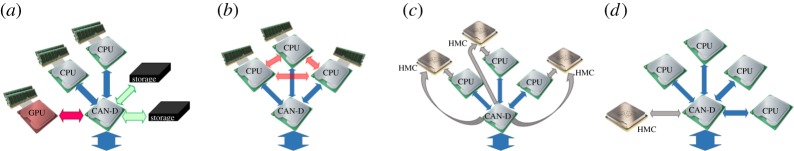
The flexibility of CAN architecture: examples of several different implementation schemes. (*a*) Centralized network fabric, where all intra-server communication is through CAN-D. (*b*) Distributed network fabric, where only part of the intra-server communication is through CAN-D. (*c*) A multi-controller memory implementation, where both CAN-D and CPU devices are attached to the RAM. (*d*) A centralized memory topology, where all devices access the RAM through CAN-D. The number and type of devices connected to CAN-D vary as well. (Online version in colour.)

CAN is an approach to computer architecture, rather than a set definition. As such, the implementation of CAN is flexible. Figure 4 illustrates several different types of implementations of CAN: centralized and distributed network fabric implementations, a centralized memory implementation and an implementation that enables multiple controllers access to memory. This flexibility makes CAN complementary to the data-centre disaggregation trend, as it can operate both as an aggregated and a disaggregated box. CAN uses an interface oblivious design that enables a single CAN-D device with flexible configurations: any single interface may operate as a compute-interconnect, a storage-interconnect or as a network-interconnect. Such flexibility is feasible today, but is typically restricted to a switching hierarchy with several different network types. This flexibility also enables the use of different networking infrastructure for CAN-D: from a standard electrical device to an experimental photonic-based fabric.

An interface-oblivious design based upon a photonic fabric and photonic-based interconnects enables scalability in a different dimension: once a CAN box is in operation, it may continuously scale. The optical switch and pathway infrastructure are oblivious to the data rate carried. Thus, a move from a 10 Gbps to 100 Gbps to 1 Tbps per port is a transparent change. The CPU may be upgraded but the I/O interface remains the same speed oblivious of the optical interconnect. This also offers possibilities to reduce costs, and ease the maintenance and upgrade processes.

The CAN architecture is the answer to computing as required by an infrastructure. This is not only because of its high performance and ability to terminate terabits, but also because of the things that matter for infrastructure: scalability, cost, reliability and management. A small CAN-based computer can serve as a personal data centre, a CAN box will provide a cloudlet's local computing infrastructure, and a collection of CAN blades will build the next-generation rackscale-based data centre.

## 7. Conclusion

We propose a new architecture for affordable HPCs, using networking practices to bridge the performance gap between the networking and the computing world. This approach is a rethinking of current models; it uses resource control commonly available within networking devices to improve both the performance and efficiency of current computers. An architecture that places networking at the centre of the machine requires a fusion of knowledge from computer systems, network design, operating systems and applications; yet it will provide for a revolution, solving issues forced on servers by current approaches and architectures.

An important part of the success of novel computing architectures has always been a combination of hardware and software contributions. CAN aims to create a close integration between hardware and software, which is essential in order to capitalize on the features provided by the hardware. On the other hand, wide adoption requires as small a number of changes as possible to the application level, otherwise adapting the applications would become too costly (in terms of time and resources). We foresee a prototype of the CAN architecture. We would envisage this based on the NetFPGA SUME [48] open source reconfigurable platform, using the CHERI [49] soft core processor and operating and extending the FreeBSD operating system.

We define our architecture as a call-to-arms, not as the definitive answer to the capacity-crunch in the end-systems but as an enabling force that will mean we can explore practical realization of novel data-centre uses: ones that are optimized to preserve our privacy through disaggregation (§2), an approach that enables a new class of use-cases enabling effective mobile devices [11], and as a practical approach to the disaggregated data centre that can both meet our future computational needs and capitalize on the disaggregated energy sources that a large-scale move to renewables (e.g. [50]) would entail. However, an architecture alone is not enough to achieve our goals. We foresee a rise in such innovative architectures also enabling new efforts to realize appropriate operating system and application innovations and believe that, irrespective of the absolute correctness of our architecture for every purpose, the innovation opportunities enabled will also have long-lasting consequences.

## Footnotes

1 For the work of CAN, we focus our discussion on commodity devices; architectures for HPCs do use more exotic interconnects, e.g. IBM's Blue Gene Q interconnect [39].

## Data accessibility

The data used to generate figure 2 are available at https://www.repository.cam.ac.uk/handle/1810/250409.
